# Multiscale Transcriptomic and Molecular Modeling Analyses Suggest DCPMU-Associated Epithelial Remodeling and Tumor Microenvironment Perturbation in Prostate Cancer

**DOI:** 10.3390/cimb48090935

**Published:** 2026-09-13

**Authors:** Jianhao Lin, Dajun Fang, Li Ding, Deqi Su

**Affiliations:** Key Laboratory of Special Environment and Health Research in Xinjiang, School of Public Health, Xinjiang Medical University, Urumqi 830017, China

**Keywords:** 3-(3,4-Dichlorophenyl)-1-methylurea, prostate cancer, network toxicology, molecular docking, single-cell transcriptomics, virtual disturbance

## Abstract

DCPMU, a major demethylated metabolite of diuron, is an environmentally persistent contaminant with potential endocrine-disrupting and carcinogenic relevance, yet its mechanistic association with prostate cancer remains unclear. This study investigated DCPMU-related mechanisms in prostate cancer by integrating network toxicology, molecular modeling, bulk transcriptomics, single-cell and spatial transcriptomics, and virtual perturbation analysis. Differentially expressed genes from TCGA-PRAD were intersected with DCPMU-associated and prostate cancer-related targets. Functional enrichment, machine learning screening, and external GEO validation identified seven candidate hub genes: APOBEC3G, SCGB1A1, PTGS1, CA12, CES1, FOLH1, and NOS1. Molecular docking showed favorable DCPMU binding to these proteins, and molecular dynamics simulations further supported stable interactions with PTGS1, CA12, CES1, and FOLH1. Single-cell analysis of GSE141445 revealed cell-type-specific expression, with FOLH1 and CA12 enriched in epithelial cells, CES1 in fibroblasts, and PTGS1 in mast cells. Spatial transcriptomics from GSE181294 showed tumor-enriched FOLH1 expression and increased Core4 module activity in malignant regions. Virtual knockout analysis suggested that FOLH1 perturbation was associated with predicted downstream transcriptional changes, with PTGS1 showing marked perturbation responsiveness. Overall, our integrative analyses suggest that DCPMU exposure is associated with epithelial malignancy, inflammatory metabolism, xenobiotic handling, and tumor-microenvironment remodeling, particularly within malignant epithelial niches. These hypothesis-generating findings require experimental validation to establish any causal role in prostate cancer progression.

## 1. Introduction

3-(3,4-Dichlorophenyl)-1-methylurea (DCPMU), also known as monomethyldiuron, is a major demethylated metabolite of diuron, a phenylurea herbicide widely used in agricultural and non-crop settings. Because diuron is extensively applied and frequently detected in environmental matrices, DCPMU should be considered an environmentally relevant exposure product rather than a negligible transient intermediate [1]. Environmental monitoring studies have detected DCPMU in surface water, groundwater, soil, and porewater, indicating its environmental persistence and potential for real-world exposure [2]. Toxicokinetic evidence further indicates that diuron undergoes substantial in vivo metabolism, with DCPMU representing a biologically relevant transformation product in human-relevant systems. Notably, DCPMU has been identified in human liver microsomal and placental models, suggesting that its formation may be relevant to human exposure scenarios [3]. Although the precise rate of diuron conversion to DCPMU in humans remains unclear, current evidence supports DCPMU as a key metabolite rather than a minor by-product. Toxicological studies have raised concerns regarding this chemical family: diuron induces urothelial tumors in rats at high dietary doses, whereas DCPMU and related metabolites exhibit endocrine-disrupting activities, including anti-androgenic and estrogenic effects in vivo [4]. These findings are significant because endocrine-active contaminants may disrupt hormone-dependent tissues before overt carcinogenic phenotypes become evident.

Prostate cancer represents a major and increasing global health burden. According to GLOBOCAN 2022, prostate cancer was the fourth most commonly diagnosed cancer worldwide and the second most common malignancy among men, with 1,467,854 new cases and 397,430 deaths globally; the corresponding age-standardized incidence and mortality rates were 29.4 and 7.3 per 100,000, respectively [5]. This substantial and increasing disease burden underscores the need to identify modifiable environmental factors that may contribute to prostate carcinogenesis. Epidemiological evidence suggests that diuron exposure may be associated with an increased risk of cancer. Using an environment-wide association study approach, Soerensen et al. evaluated county-level use of 295 pesticides in the continental United States in relation to prostate cancer outcomes. Among these pesticides, 22, including diuron, showed consistent and significant positive associations with prostate cancer incidence in both the discovery and validation cohorts. In the validation cohort, each one-standard-deviation increase in log-transformed diuron use was associated with an increase of 3.27 prostate cancer cases per 100,000 individuals (95% CI: 1.99–4.55) [6]. In addition, a cross-sectional study involving 10,646 farmers in Nakhon Sawan, Thailand, reported a trend toward an increased overall cancer risk among diuron users (adjusted OR = 1.46, 95% CI: 0.63–3.37), although the association did not reach statistical significance [7]. Collectively, these findings suggest that diuron may pose a potential carcinogenic risk. However, because the available evidence is primarily derived from ecological and cross-sectional association studies, causal relationships remain to be established through individual-level exposure assessments and experimental validation.

An integrative multi-omics strategy is therefore required to systematically dissect the potential effects of DCPMU on prostate cancer. Network toxicology can be used to predict candidate targets and signaling pathways potentially associated with DCPMU, whereas single-cell RNA sequencing can resolve cell type-specific transcriptional alterations [8], and spatial transcriptomics can preserve tissue architecture to reveal spatially organized niches and patterns of intercellular communication [9]. In the present study, we therefore integrated network toxicology, single-cell transcriptomics, spatial transcriptomics, and in silico virtual knockout analysis to investigate the potential molecular associations between DCPMU and prostate cancer. This approach aimed to identify key genes, pathways, and cellular populations involved in the putative biological effects of DCPMU.

## 2. Materials and Methods

**Figure 1:**
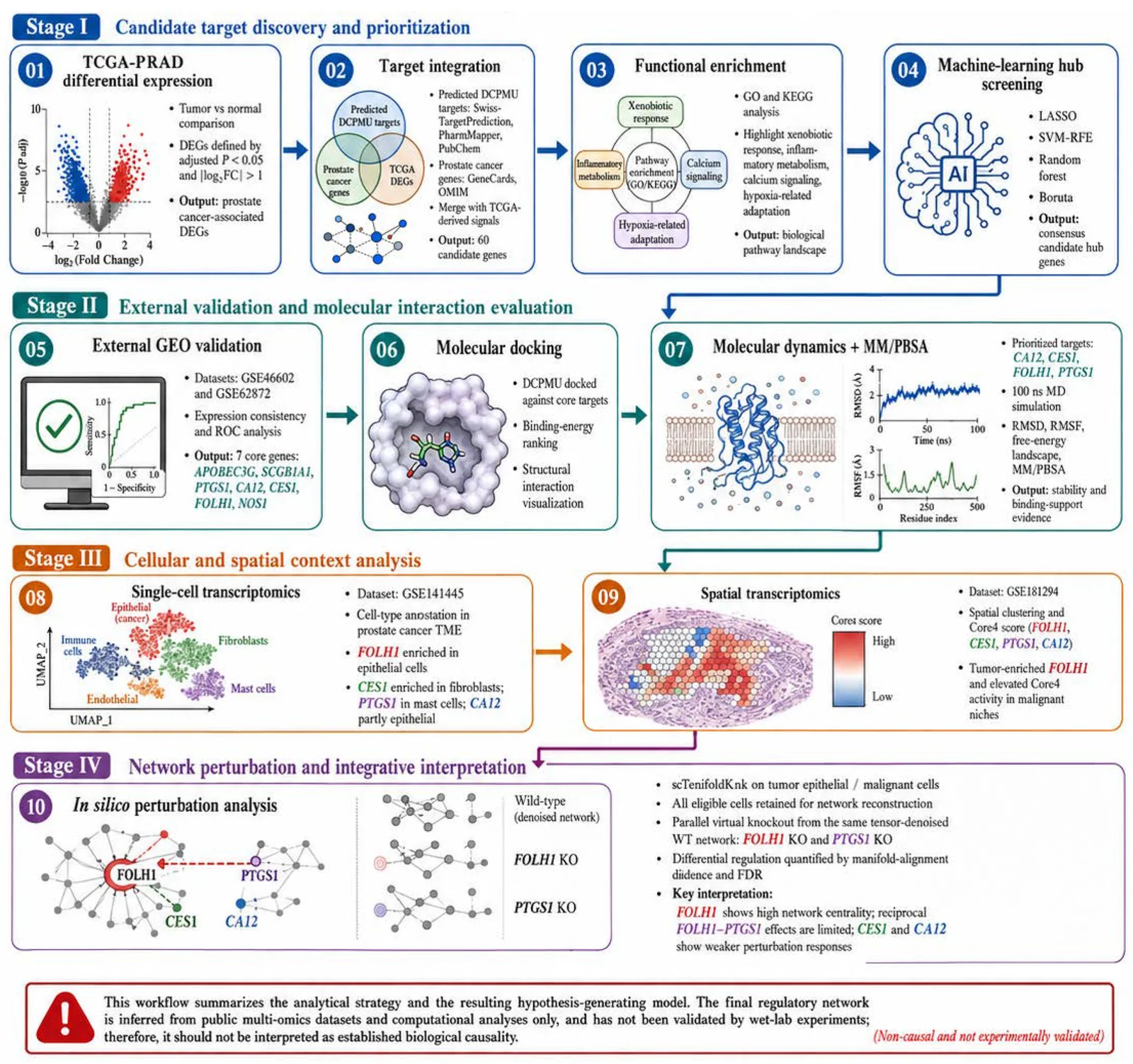
Path diagram of this study.

Figure 1, created with BioGDP.com [10], demonstrates the specific workflow of this study.

### 2.1. Differential Expression Analysis of TCGA-PRAD

Differential expression analysis between prostate cancer tissues and normal prostate tissues in the TCGA-PRAD dataset was performed using the R package limma (version 3.68.5), depending on the format of the downloaded data [11]. Genes with adjusted *p* < 0.05 and |log2 fold change (FC)| > 1 were considered differentially expressed. Heatmaps of the top differentially expressed genes and volcano plots of all genes were generated using the pheatmap (version 1.0.13) and ggplot2 (version 4.0.2) packages, respectively [12].

### 2.2. Integration of DCPMU-Related Targets, Prostate Cancer-Associated Genes, and TCGA-Derived DEGs

Potential DCPMU targets were retrieved from public chemical-target prediction and toxicology databases, including SwissTargetPrediction (http://www.swisstargetprediction.ch/ accessed on 30 April 2026), PharmMapper Version 2017 (http://lilab-ecust.cn/pharmmapper/ accessed on 30 April 2026), and PubChem (https://pubchem.ncbi.nlm.nih.gov/ accessed on 30 April 2026). After merging the results, duplicate entries were removed, and gene symbols were standardized using the UniProt database [13]. Prostate cancer-related genes were collected from disease-associated databases, including GeneCards and OMIM. After duplicate removal, a comprehensive prostate cancer-related target set was established. Finally, the predicted DCPMU targets, prostate cancer-related genes, and differentially expressed genes (DEGs) were intersected to identify potential DCPMU-associated target genes in prostate cancer.

### 2.3. Intersection Analysis and Functional Enrichment Analysis

Gene Ontology (GO) enrichment analysis, comprising biological process (BP), cellular component (CC), and molecular function (MF) categories [14], together with Kyoto Encyclopedia of Genes and Genomes (KEGG) pathway enrichment analysis [15], was performed using the clusterProfiler R package (version 4.18.4). Terms with an adjusted *p*-value < 0.05 were considered significantly enriched.

### 2.4. Machine Learning-Based Identification of Candidate Hub Genes

Candidate genes were evaluated for distinguishing primary prostate tumors from normal tissues in TCGA-PRAD. Log2(TPM + 1)-transformed data were divided into stratified training and test sets at a 7:3 ratio. Feature selection and model optimization were performed only in the training set. LASSO used 10-fold cross-validation and retained genes with non-zero coefficients at lambda.1se [16]. SVM-RFE and random forest were optimized using repeated five-fold cross-validation with three repeats and ROC AUC as the performance metric; the random forest contained 1000 trees [17,18]. Boruta was performed with up to 300 iterations to identify confirmed features [19]. Model performance was assessed in the independent test set using ROC AUC. Genes identified by at least three of the four algorithms were retained as candidate hub genes for external validation.

### 2.5. External Validation of Candidate Hub Genes in GEO Datasets

The expression patterns of candidate hub genes were independently validated using two external prostate cancer cohorts, GSE46602 and GSE62872, obtained from the Gene Expression Omnibus (GEO) database. Differential expression analysis between prostate cancer and normal control samples was performed using the limma package for microarray datasets. For genes with non-normally distributed expression values, appropriate nonparametric statistical tests were applied. Genes showing consistent expression alterations between tumor and control groups in both independent GEO cohorts were considered robustly validated candidates. Furthermore, receiver operating characteristic (ROC) curve analysis was conducted using the pROC package (version 1.19.0.1) to evaluate the diagnostic performance of these validated genes, with the area under the curve (AUC) calculated to quantify their discriminative ability between prostate cancer and normal tissues.

### 2.6. Molecular Docking Analysis

Molecular docking was used to explore the potential binding of DCPMU to core targets prior to prostate cancer. AutoDock Vina (version 1.2.7) was employed for docking [20], and the results of docking were visualized using PyMOL (version 2.6). The retrieved chemical formulae and core genes were converted to pdb format files using Open Babel (version 3.1.1) and then docked using AutoDock Vina; the docking results with low binding energy and all three chemical components were selected and visualized using PyMOL [21].

### 2.7. Molecular Dynamics Simulation

We conducted molecular dynamics (MD) simulations on the top four genes with the lowest molecular docking ability using GROMACS 2022 software [22]. Ligand parameters were obtained from the CGenFF online server, and hydrogen atoms were added using Avogadro. Protein-ligand systems were parameterized with the CHARMM36 force field. Energy minimization was performed using the steepest descent algorithm, followed by 100 ps NVT equilibration and 100 ps NPT equilibration, with a 2 fs integration time step. The temperature was maintained at 310 K using the V-rescale thermostat, and the pressure was maintained at 1 bar using the Parrinello–Rahman barostat. Long-range electrostatic interactions were computed using the particle mesh Ewald (PME) method, with short-range electrostatic and van der Waals interactions truncated at 1.0 nm. Hydrogen-containing bonds were constrained using the LINCS algorithm, and periodic boundary conditions were applied in all three dimensions. Production MD simulations were then run for 100 ns. After the MD simulations, built-in tools in GROMACS 2022 were used to calculate properties of the complexes, including root-mean-square deviation (RMSD), root-mean-square fluctuation (RMSF), and Gibbs free energy profiles. Binding free energies for the CA12, CES1, FOLH1, and PTGS1 complexes were estimated using the MM/PBSA approach based on MD trajectories. Frames were extracted from the 80–90 ns interval, which was used as the production sampling window, and processed using MMPBSA to calculate the binding free energies.

### 2.8. Single-Cell Sequencing Analysis

Publicly available single-cell RNA-sequencing data from GSE141445 were analyzed using Seurat [23]. Cells with fewer than 200 or more than 6000 detected genes or with mitochondrial transcript proportions above 20% were excluded. Data were normalized using the LogNormalize method, and 3000 highly variable genes were selected using the variance-stabilizing transformation method. After scaling, principal component analysis was performed, and the first 30 principal components were used for shared nearest-neighbor clustering at a resolution of 0.8 and UMAP visualization. No additional batch-effect correction was applied. Cluster markers were identified using FindAllMarkers with only.pos = TRUE, min.pct = 0.25, and logfc.threshold = 0.25. Cell types were annotated according to canonical lineage markers and module scores calculated using AddModuleScore; cells with a difference of less than 0.05 between the two highest scores were classified as ambiguous. The expression of FOLH1, CES1, PTGS1, and CA12 across annotated cell populations was evaluated using dot plots, UMAP feature plots, violin plots, and cell-type-specific average expression values.

### 2.9. Spatial Transcriptomic Analysis

Spatial transcriptomic data from GSE181294 were analyzed using Seurat. Spots with fewer than 100 detected genes or fewer than 200 total transcript counts were excluded. The merged expression matrix was normalized using the LogNormalize method with a scale factor of 10,000, and 3000 highly variable genes were selected using the variance-stabilizing transformation method. After scaling and principal component analysis, up to 30 principal components were used for UMAP visualization, nearest-neighbor construction, and clustering at a resolution of 0.4. Cluster-enriched genes were identified using FindAllMarkers with only.pos = TRUE, min.pct = 0.25, and a log2 fold-change threshold of 0.25. The Core4 signature was predefined as the four DCPMU-associated genes FOLH1, CES1, PTGS1, and CA12. A composite module score, termed Core4_Score1, was calculated for each spatial spot using Seurat’s AddModuleScore function based on all detectable Core4 genes. Higher Core4_Score1 values indicate relative coordinated enrichment of this four-gene expression signature compared with matched background control genes, rather than direct pathway activation or a causal biological effect. The spatial distribution and sample-level mean of the Core4 score were compared across healthy, benign, and tumor samples to evaluate its enrichment in tumor-associated spatial regions [24].

### 2.10. In Silico Perturbation Analysis

Target cells were extracted from the normalized single-cell expression matrix and visualized using UMAP. FOLH1 expression was mapped onto the embedding to identify FOLH1-enriched prostate epithelial/tumor-associated cells. All eligible tumor epithelial/malignant cells were retained for network reconstruction, and each eligible cell was guaranteed to contribute to at least one gene regulatory network (GRN). The analysis included 3000 highly variable genes together with FOLH1, PTGS1, CES1, and CA12, while genes expressed in fewer than 25 cells were excluded, except for prespecified candidate genes retained for downstream analysis. Multiple GRNs were constructed using predefined cell subsets and integrated by CP tensor decomposition to generate a common denoised WT regulatory network. Virtual knockout of FOLH1 and PTGS1 was then performed in parallel from the same common WT network by removing the outgoing regulatory edges of the corresponding knockout gene. The WT and knockout networks were aligned using manifold alignment, and differential regulation was quantified using manifold-alignment distances and false-discovery-rate-adjusted *p*-values [25]. FOLH1, PTGS1, CES1, and CA12 were specifically highlighted to assess reciprocal and cross-knockout regulatory responses. These analyses were designed to characterize network-level perturbation rather than directional changes in gene expression.

## 3. Results

### 3.1. Differential Expression Analysis in the TCGA-PRAD Dataset

**Figure 2:**
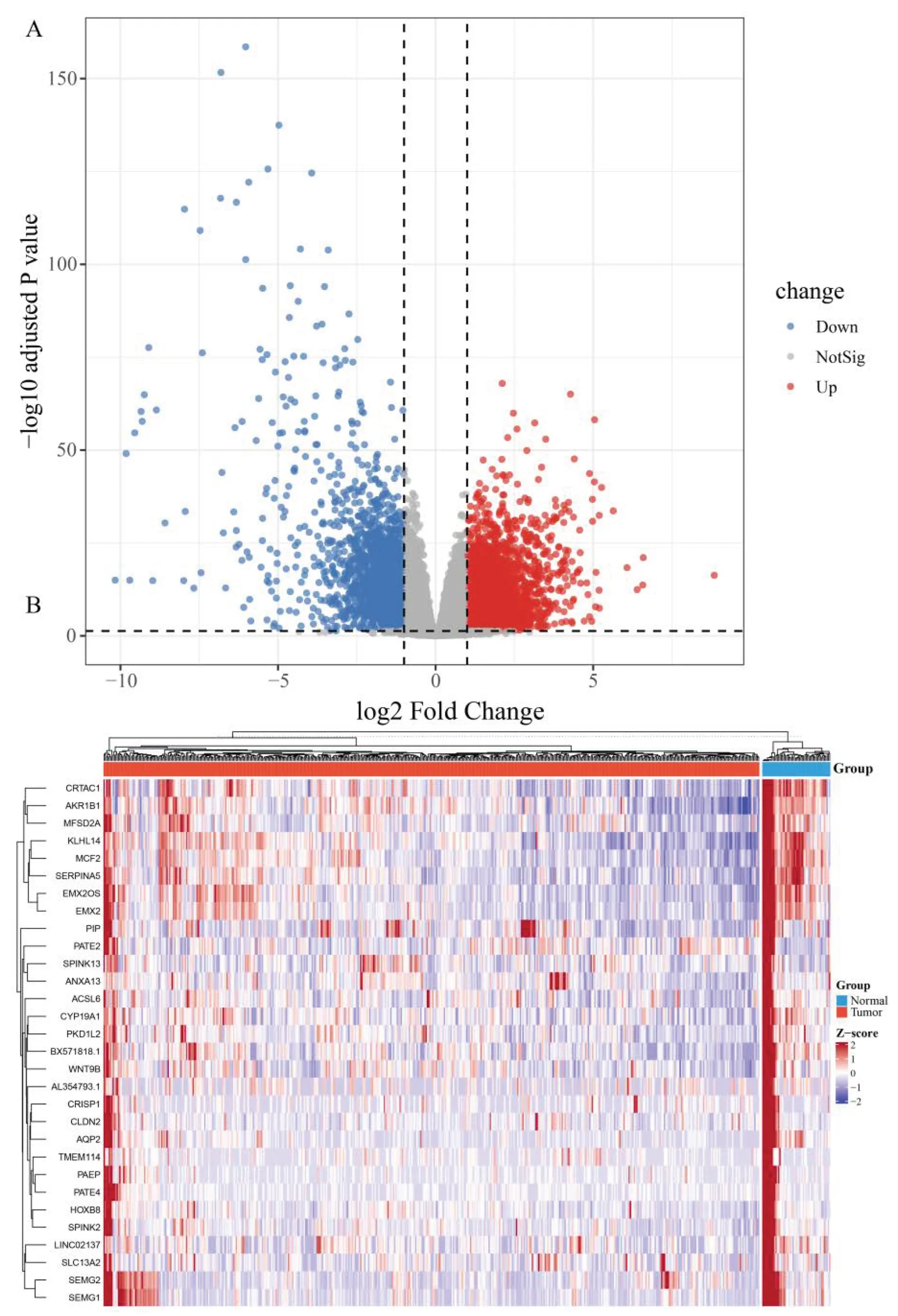
Differential expression analysis of TCGA-PRAD: (**A**) Heatmap showing the expression profiles of differentially expressed genes between normal and tumor samples. (**B**) Volcano plot showing the upregulated, downregulated, and non-significant genes in TCGA-PRAD. Red dots represent upregulated genes, blue dots represent downregulated genes, and gray dots represent genes without significant differential expression. The vertical dashed lines indicate the log2 fold-change thresholds, while the horizontal dashed line indicates the statistical significance threshold.

Differential expression analysis of the TCGA-PRAD dataset revealed pronounced transcriptomic differences between prostate cancer and normal prostate tissues. As shown in Figure 2A, a large number of genes were significantly upregulated or downregulated in prostate cancer, suggesting extensive transcriptional reprogramming during tumor development. The heatmap in Figure 2B further showed that representative differentially expressed genes displayed distinct expression patterns between tumor and normal tissues, allowing broad separation of the two groups. Overall, these findings indicate that prostate cancer tissues exhibit substantial transcriptomic alterations compared with normal prostate tissues, thereby providing a foundation for subsequent target intersection analysis and hub gene screening.

### 3.2. Identification of an Integrated DCPMU-Associated Candidate Gene Set in Prostate Cancer

**Figure 3:**
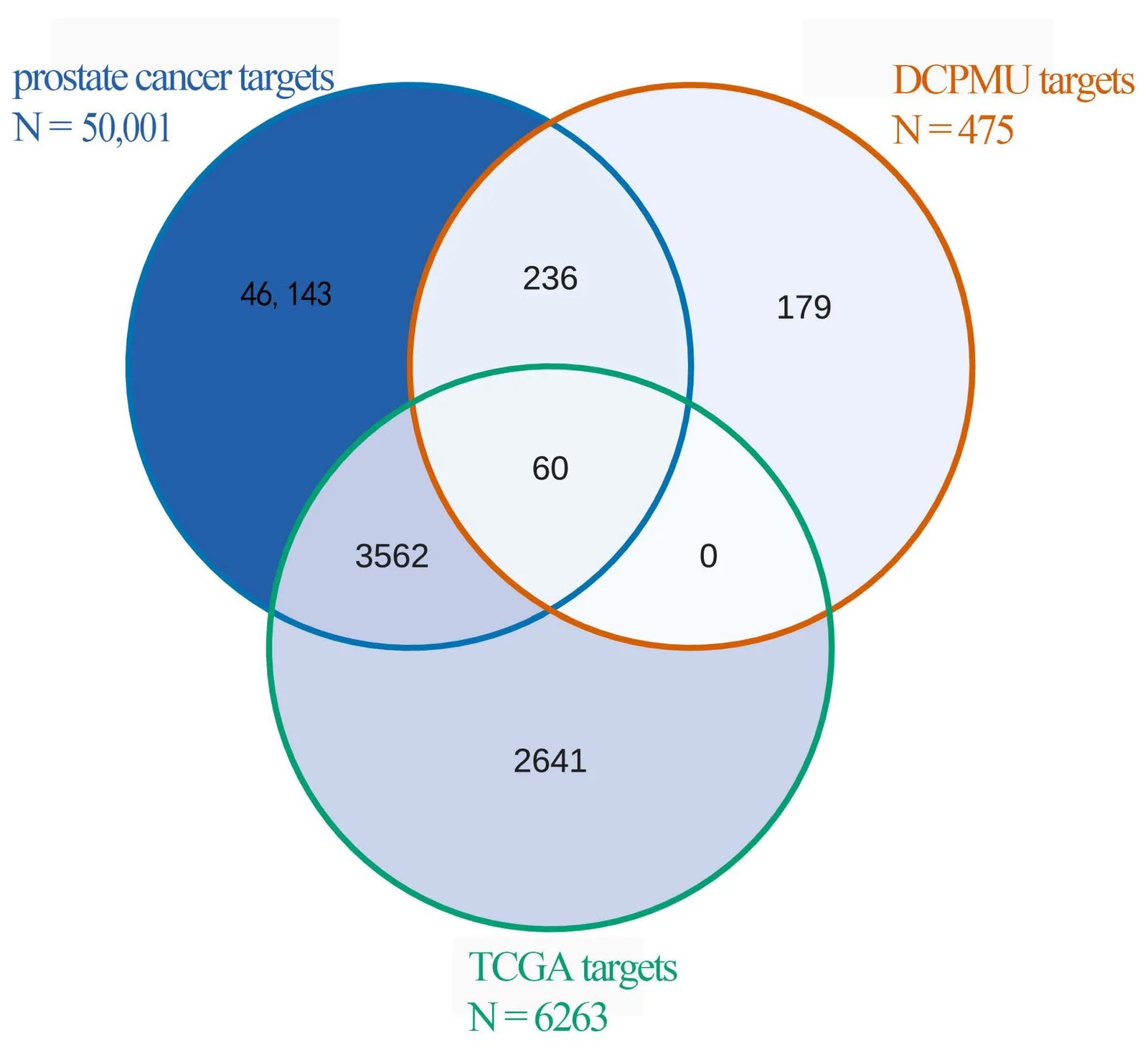
Identification of candidate DCPMU-related targets in prostate cancer. Venn diagram showing the overlap among prostate cancer–associated targets (*n* = 50,001), predicted DCPMU targets (*n* = 475), and TCGA-derived prostate cancer-related targets (*n* = 6263). A total of 60 shared targets were identified for subsequent analyses.

To comprehensively identify candidate genes potentially involved in DCPMU-associated prostate cancer, DCPMU-related targets, prostate cancer-associated genes retrieved from public databases, and differentially expressed genes (DEGs) from the TCGA-PRAD dataset were integrated using a set-union strategy. As shown in Figure 3, this analysis yielded 60 nonredundant genes, including APOBEC3G, AURKB, CA12, CA14, CES1, CXCR2, DMC1, FOLH1, GCK, NOS1, PTGS1, SCGB1A1, and SOX5. By incorporating both disease-associated genes and DCPMU-related targets, this integrated gene set expanded the candidate target space and reduced the risk of excluding biologically relevant genes that might not satisfy strict intersection criteria. These 60 genes were subsequently subjected to functional enrichment analysis and machine learning-based feature selection.

### 3.3. Functional Enrichment Analysis of Candidate Genes

**Figure 4:**
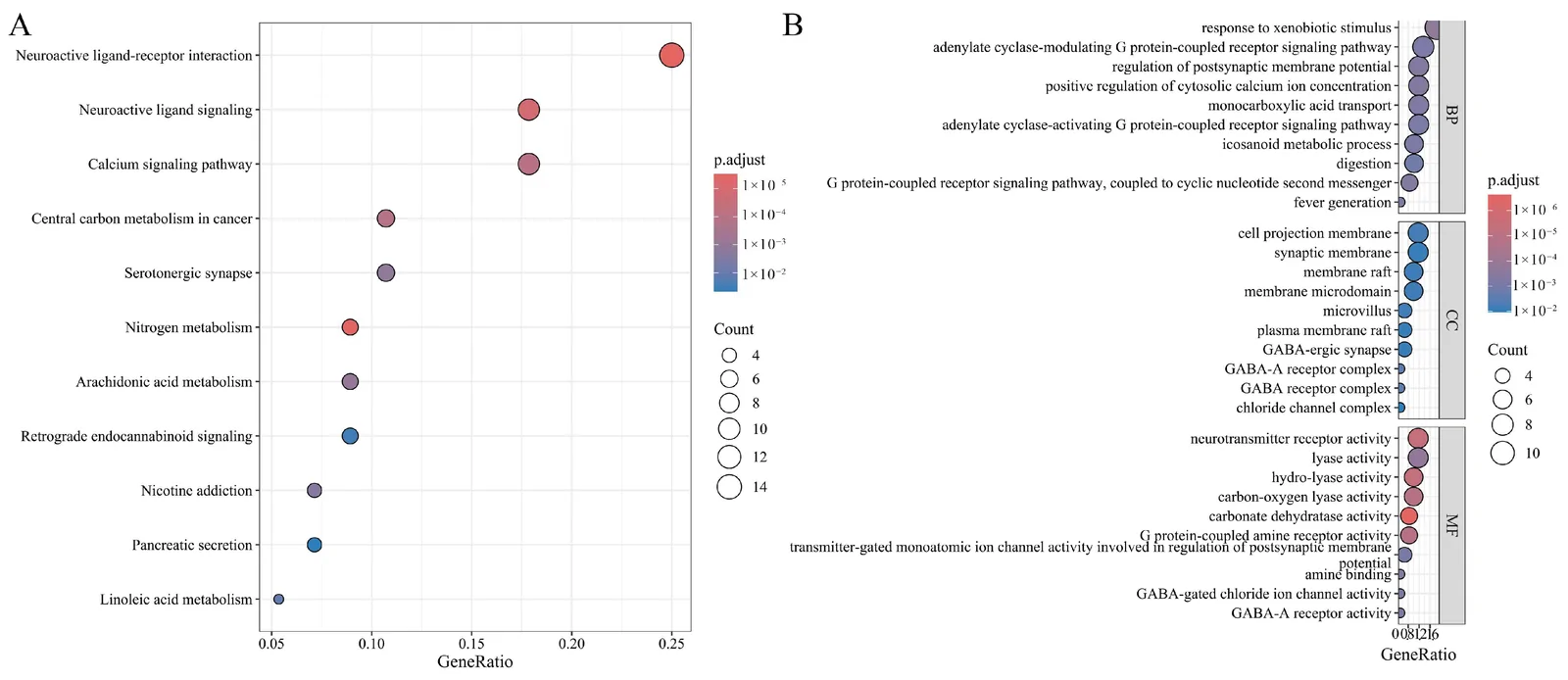
KEGG and GO enrichment results for prostate cancer (**A**,**B**) depict the distribution of prostate cancer-associated genes across biological process (BP), molecular function (MF), cellular component (CC), and KEGG pathway categories. The length of each bar is proportional to the number of genes enriched in a given term or pathway, and the bar color reflects the *p*-value, with redder colors indicating smaller *p*-values and stronger enrichment for that function or pathway.

As shown in Figure 4A, KEGG pathway analysis revealed significant enrichment of these genes in neuroactive ligand-receptor interaction, calcium signaling, central carbon metabolism in cancer, serotonergic synapse, nitrogen metabolism, arachidonic acid metabolism, retrograde endocannabinoid signaling, and linoleic acid metabolism. Among these pathways, neuroactive ligand-receptor interaction showed the strongest enrichment, suggesting that dysregulated receptor-mediated signaling may represent a major functional characteristic of the candidate gene set. Collectively, these findings indicate that the identified genes are mainly involved in signal transduction, neurotransmitter-related signaling, calcium homeostasis, and metabolic reprogramming, which may contribute to prostate cancer development and progression. As shown in Figure 4B, GO enrichment analysis indicated that these genes were mainly associated with biological processes related to response to xenobiotic stimulus, adenylate cyclase-modulating or activating G protein-coupled receptor signaling, regulation of postsynaptic membrane potential, positive regulation of cytosolic calcium ion concentration, and icosanoid metabolic processes. In the cellular component category, the enriched genes were primarily localized to the cell projection membrane, synaptic membrane, membrane raft, microvillus membrane, and GABA receptor complex. In the molecular function category, the major enriched terms included neurotransmitter receptor activity, lyase activity, hydrolyase activity, carbonate dehydratase activity, G protein-coupled amine receptor activity, and GABA-A receptor activity.

### 3.4. Consensus Candidate Hub Gene Identification Using Multiple Machine Learning Algorithms

**Figure 5:**
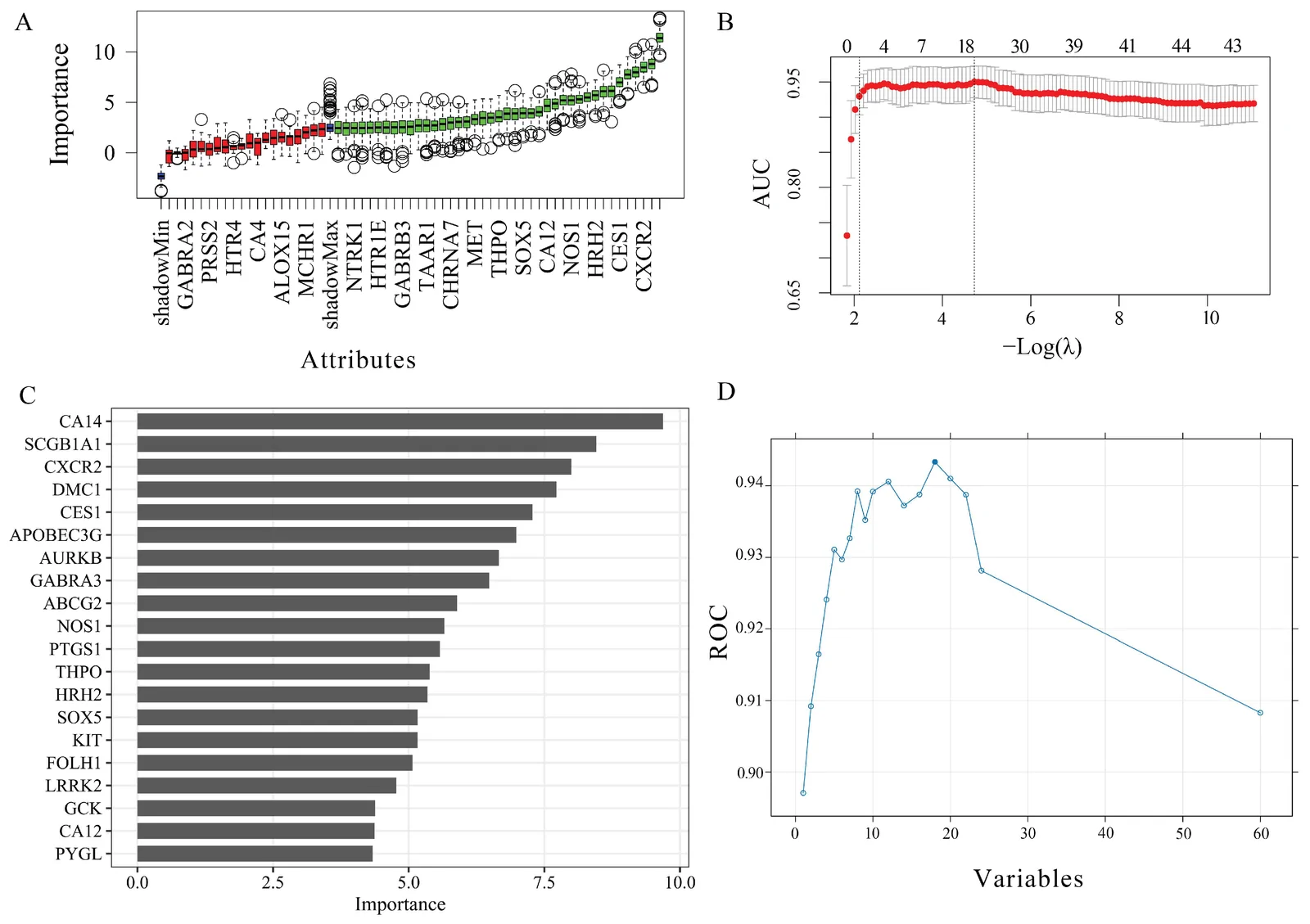
Machine learning-based feature selection for identifying key candidate genes: (**A**) Feature selection using the least absolute shrinkage and selection operator (LASSO) regression model. Green indicates confirmed important features, red indicates rejected features, and blue represents shadow attributes. (**B**) Feature screening using support vector machine-recursive feature elimination (SVM-RFE). (**C**) Variable importance ranking generated by the random forest model. (**D**) Confirmation of important features using the Boruta algorithm.

To further refine the candidate gene set, four machine learning algorithms, namely LASSO, SVM-RFE, random forest, and Boruta, were applied to the 60 merged candidate genes obtained from the union analysis. The corresponding results are shown in Figure 5A–D. LASSO regression retained two genes, SVM-RFE identified 18 genes with the best classification performance, random forest selected the top 20 genes based on variable importance, and Boruta identified 41 relevant features. Among these algorithms, SVM-RFE achieved the highest classification performance when 18 variables were included, with a cross-validated ROC-AUC of approximately 0.94. Feature importance analysis showed that CA14, SCGB1A1, CXCR2, DMC1, CES1, APOBEC3G, AURKB, and FOLH1 ranked among the most informative genes, indicating their strong contributions to distinguishing prostate cancer from normal samples. Integrative comparison across the four algorithms identified 14 consensus genes selected by at least three methods: CA14, CXCR2, APOBEC3G, AURKB, CA12, CES1, DMC1, FOLH1, GABRA3, GCK, NOS1, PTGS1, SCGB1A1, and SOX5. Notably, CA14 and CXCR2 were consistently retained by all four algorithms, suggesting particularly robust predictive potential. These consensus genes were therefore defined as candidate hub genes and carried forward for subsequent external validation.

### 3.5. External Validation Identified Seven Core Genes in Two Independent GEO Datasets

**Figure 6:**
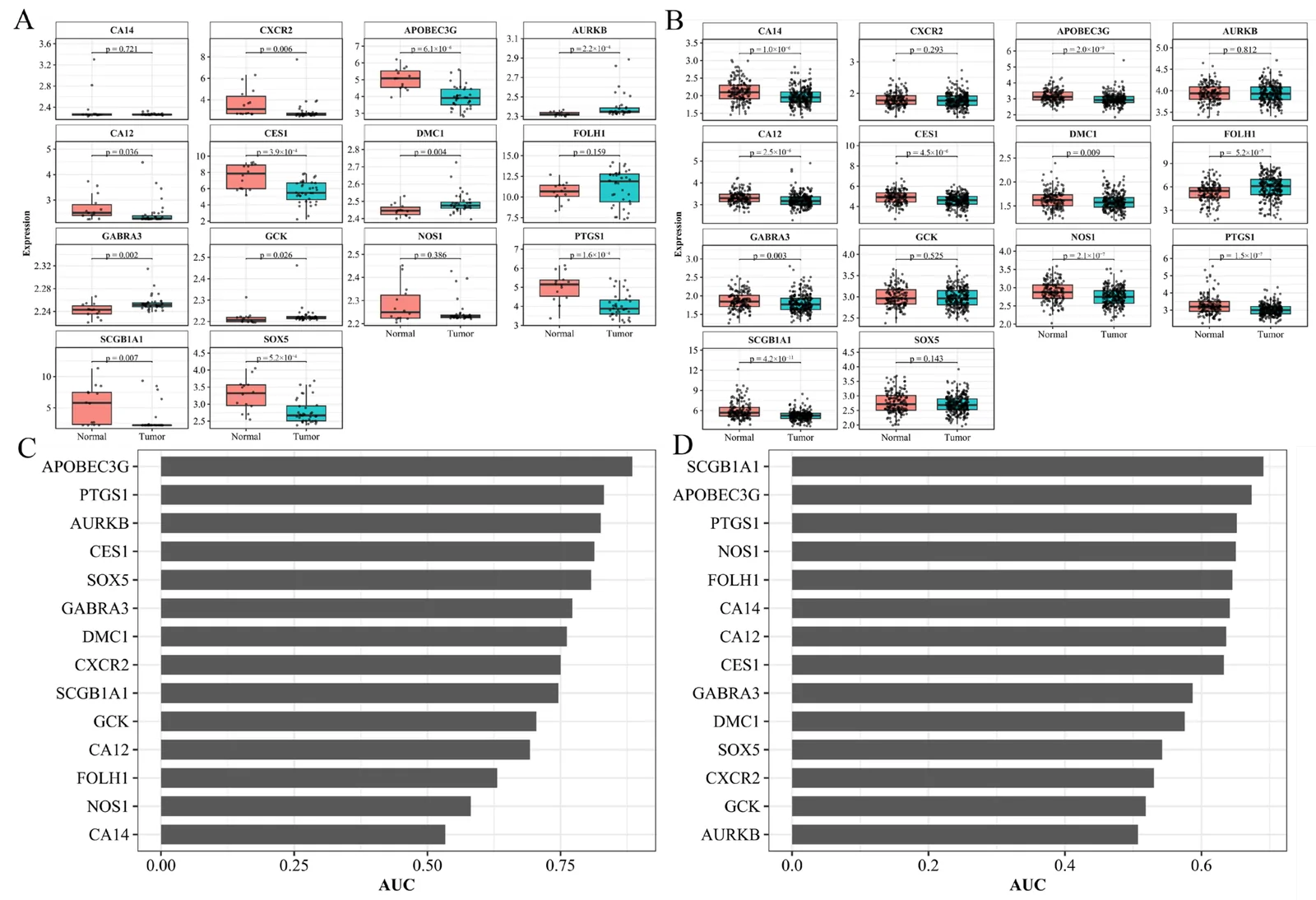
External validation of candidate genes in independent datasets: (**A**,**B**) Expression validation of the selected candidate genes between normal and tumor samples in the GSE46602 and GSE62872 datasets, respectively. (**C**,**D**) Receiver operating characteristic (ROC) curves and corresponding AUC values for individual candidate genes in the GSE46602 and GSE62872 datasets, respectively. These results support the generalizability and potential clinical utility of the identified gene signature across independent populations.

To further assess the robustness of the machine learning-derived candidate genes, external validation was performed using two independent GEO datasets, GSE46602 and GSE62872. The corresponding results are shown in Figure 6A−D. Comparative expression analysis showed that APOBEC3G, CES1, PTGS1, SCGB1A1, and CA12 were consistently downregulated in tumor tissues across both datasets, whereas FOLH1 was consistently upregulated and NOS1 showed a concordant downward trend. Based on these consistent expression patterns, APOBEC3G, CES1, PTGS1, SCGB1A1, CA12, FOLH1, and NOS1 were defined as core genes for subsequent analyses. In GSE46602, APOBEC3G, CES1, PTGS1, SCGB1A1, and CA12 were significantly differentially expressed between normal and tumor samples, with particularly pronounced differences observed for APOBEC3G (*P* = 6.1 × 10^−6^), CES1 (*P* = 3.9 × 10^−4^), and PTGS1 (*P* = 1.6 × 10^−4^). In the same dataset, FOLH1 showed an upward trend in tumors, whereas NOS1 showed a downward trend. In GSE62872, these expression patterns were largely reproduced, with significant downregulation of APOBEC3G (*P* = 2.0 × 10^−9^), CES1 (*P* = 4.5 × 10^−6^), PTGS1 (*P* = 1.5 × 10^−7^), SCGB1A1 (*P* = 4.2 × 10^−11^), CA12 (*P* = 2.5 × 10^−6^), and NOS1 (*P* = 2.1 × 10^−7^), together with significant upregulation of FOLH1 (*P* = 5.2 × 10^−7^). ROC analysis further supported the discriminatory potential of these genes. In GSE46602, APOBEC3G, PTGS1, and CES1 demonstrated relatively strong diagnostic performance, with AUC values of 0.885, 0.831, and 0.813, respectively, whereas SCGB1A1 and CA12 exhibited moderate predictive ability. In GSE62872, the diagnostic performance of individual genes was lower yet remained consistent across all seven core genes, with AUC values ranging from 0.633 to 0.691. Collectively, these findings indicate that the seven selected genes exhibit stable expression trends across independent datasets and may serve as robust prostate cancer-associated candidate core genes.

### 3.6. The Results of Molecular Docking

**Figure 7:**
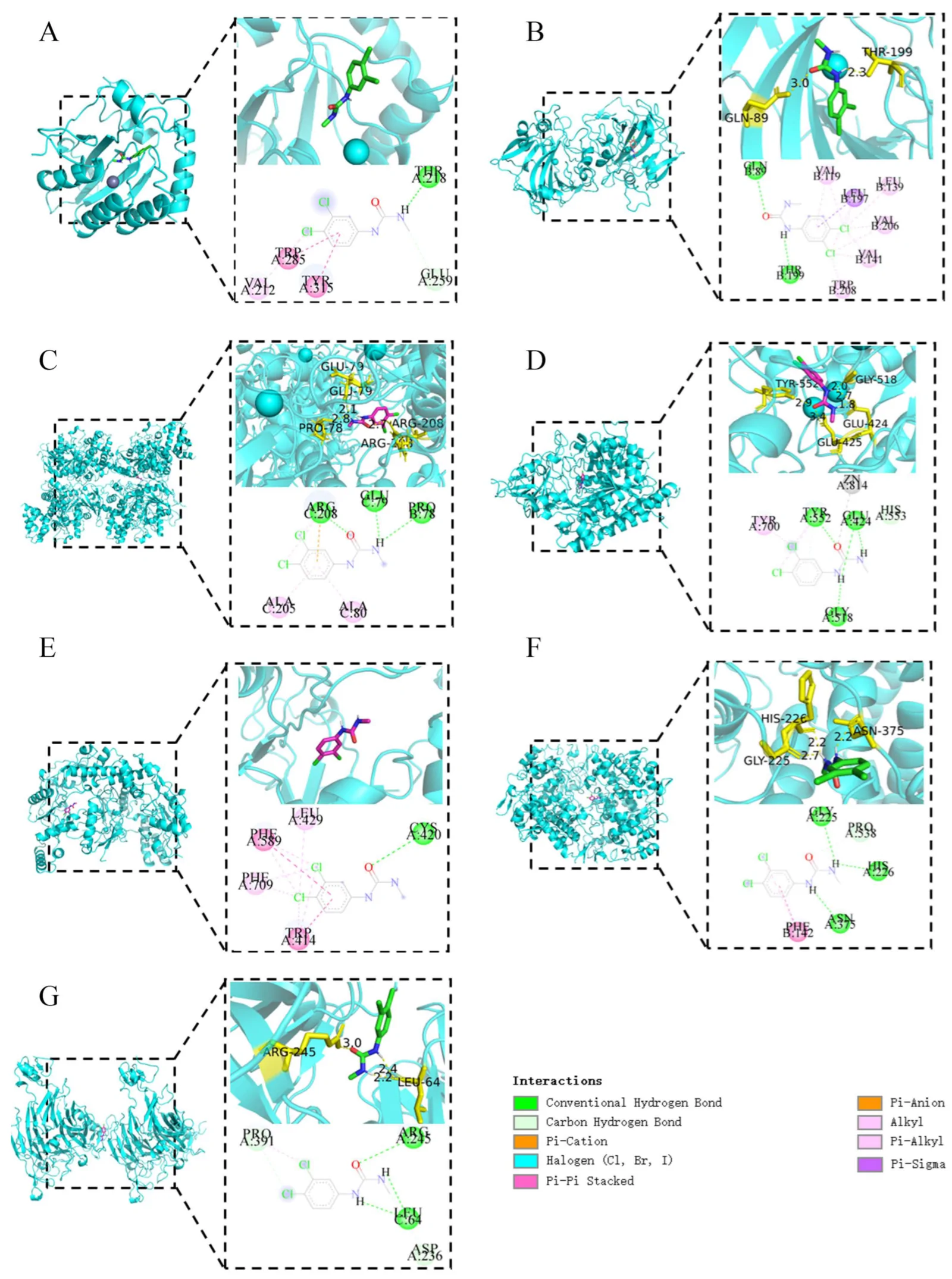
Molecular docking diagrams illustrating the lowest binding energy conformations between DCPMU and the seven target proteins: (**A**) DCPMU and SCGB1A1, (**B**) DCPMU and PTGS1, (**C**) DCPMU and NOS1, (**D**) DCPMU and FOLH1, (**E**) DCPMU and CES1, (**F**) DCPMU and CA12, and (**G**) DCPMU and APOBEC3G.

The primary metric used to evaluate interaction strength in molecular docking is the binding energy, which is typically reported as a negative value; more negative values indicate stronger predicted binding. In general, a binding energy below −5 kcal/mol is considered indicative of a stable ligand-protein interaction. Molecular docking analysis showed that all seven core proteins exhibited high predicted binding affinities toward DCPMU. The binding energies were organized in descending order of binding affinity, as outlined below: NOS1 (−7.407 kcal/mol), FOLH1 (−7.273 kcal/mol), CES1 (−7.213 kcal/mol), CA12 (−7.035 kcal/mol), PTGS1 (−6.496 kcal/mol), SCGB1A1 (−5.79 kcal/mol), and APOBEC3G (−5.536 kcal/mol). The resulting complexes and intermolecular interactions were visualized as illustrated in Figure 7A–G. Overall, these docking results suggest that DCPMU can form stable interactions with these key target proteins. These predicted interactions provide a preliminary structural basis for hypothesizing the possible molecular targets and pharmacological mechanisms of DCPMU, which warrant further experimental and functional validation.

### 3.7. The Results of Molecular Dynamics Simulations

**Figure 8:**
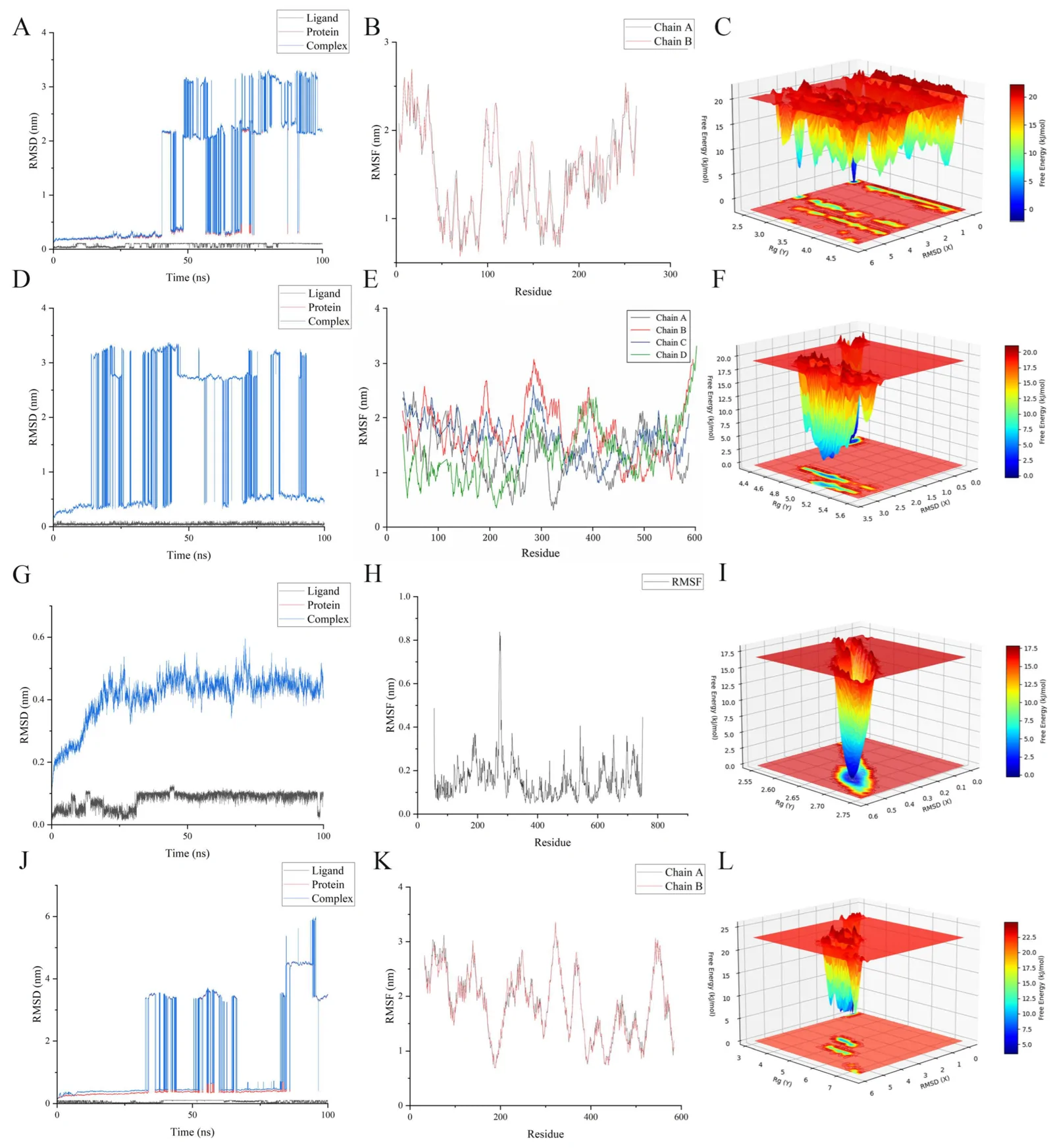
Molecular dynamics simulation results of DCPMU. RMSD of the protein–ligand complexes: (**A**) CA12, (**D**) CES1, (**G**) FOLH1, and (**J**) PTGS1. RMSF of the protein–ligand complexes: (**B**) CA12, (**E**) CES1, (**H**) FOLH1, and (**K**) PTGS1. Gibbs free energy landscape of the protein–ligand complexes: (**C**) CA12, (**F**) CES1, (**I**) FOLH1, and (**L**) PTGS1.

Molecular dynamics (MD) simulations were performed for four target proteins, CA12, CES1, FOLH1, and PTGS1, to further evaluate the stability of the corresponding DCPMU-protein complexes. Root mean square deviation (RMSD) was used to assess the overall conformational stability of each system during the simulation. As shown in Figure 8A–C, the CA12-DCPMU complex exhibited pronounced RMSD transitions after approximately 35 ns, indicating substantial conformational rearrangement and limited structural stability. Similarly, the CES1-DCPMU complex showed frequent RMSD fluctuations between low- and high-deviation states, suggesting that the system adopted metastable conformations rather than maintaining a single stable binding state (Figure 8D–F). In contrast, the FOLH1-DCPMU complex reached a stable RMSD plateau of approximately 0.4–0.5 nm after equilibration and displayed relatively low residue fluctuations and a compact low-energy basin, indicating the highest conformational stability among the four systems (Figure 8G–I). The PTGS1-DCPMU complex exhibited progressively increasing RMSD values, reaching nearly 6.0 nm toward the end of the simulation, together with a dispersed free-energy distribution, indicating extensive conformational deviation and the lowest stability (Figure 8J–L). Root mean square fluctuation (RMSF) analysis showed that the CA12-DCPMU, CES1-DCPMU, and PTGS1-DCPMU systems exhibited relatively high residue flexibility, whereas the FOLH1-DCPMU complex displayed markedly lower fluctuations, indicating greater structural rigidity. Consistently, the free-energy landscape of FOLH1-DCPMU showed a compact and deep low-energy basin, supporting a dominant stable conformation. In contrast, the other three systems exhibited broader or more dispersed energy distributions, suggesting multiple conformational states and lower thermodynamic stability. Collectively, these MD simulation results indicate that DCPMU forms stable complexes with CA12, CES1, FOLH1, and PTGS1, as evidenced by stable conformational behavior, reduced local residue flexibility near the binding pockets, and energetically favorable conformational states. These findings further support the reliability of the molecular docking predictions. As shown in Table 1, energy decomposition analysis revealed that binding was predominantly driven by favorable gas-phase interactions in CA12, FOLH1, and PTGS1, with van der Waals interactions providing the major favorable contribution, highlighting the important role of hydrophobic and dispersion contacts in complex stabilization. Among the four systems, FOLH1 exhibited the most favorable gas-phase contribution (GGAS = −185.205 kJ/mol), mainly arising from its strong van der Waals interaction (VDWAALS = −156.628 kJ/mol), together with a favorable electrostatic contribution. Although solvation opposed binding for FOLH1, CA12, and PTGS1, the favorable gas-phase interactions were sufficient to overcome the corresponding solvation penalties. FOLH1 ultimately showed the most favorable total binding free energy (−103.545 kJ/mol), indicating the strongest predicted binding affinity, followed by CA12 (−66.546 kJ/mol) and PTGS1 (−60.459 kJ/mol). In contrast, CES1 displayed unfavorable gas-phase interactions and a positive total binding free energy, suggesting substantially weaker or unfavorable binding compared with the other three systems.

### 3.8. The Results of Single-Cell Sequencing

**Figure 9:**
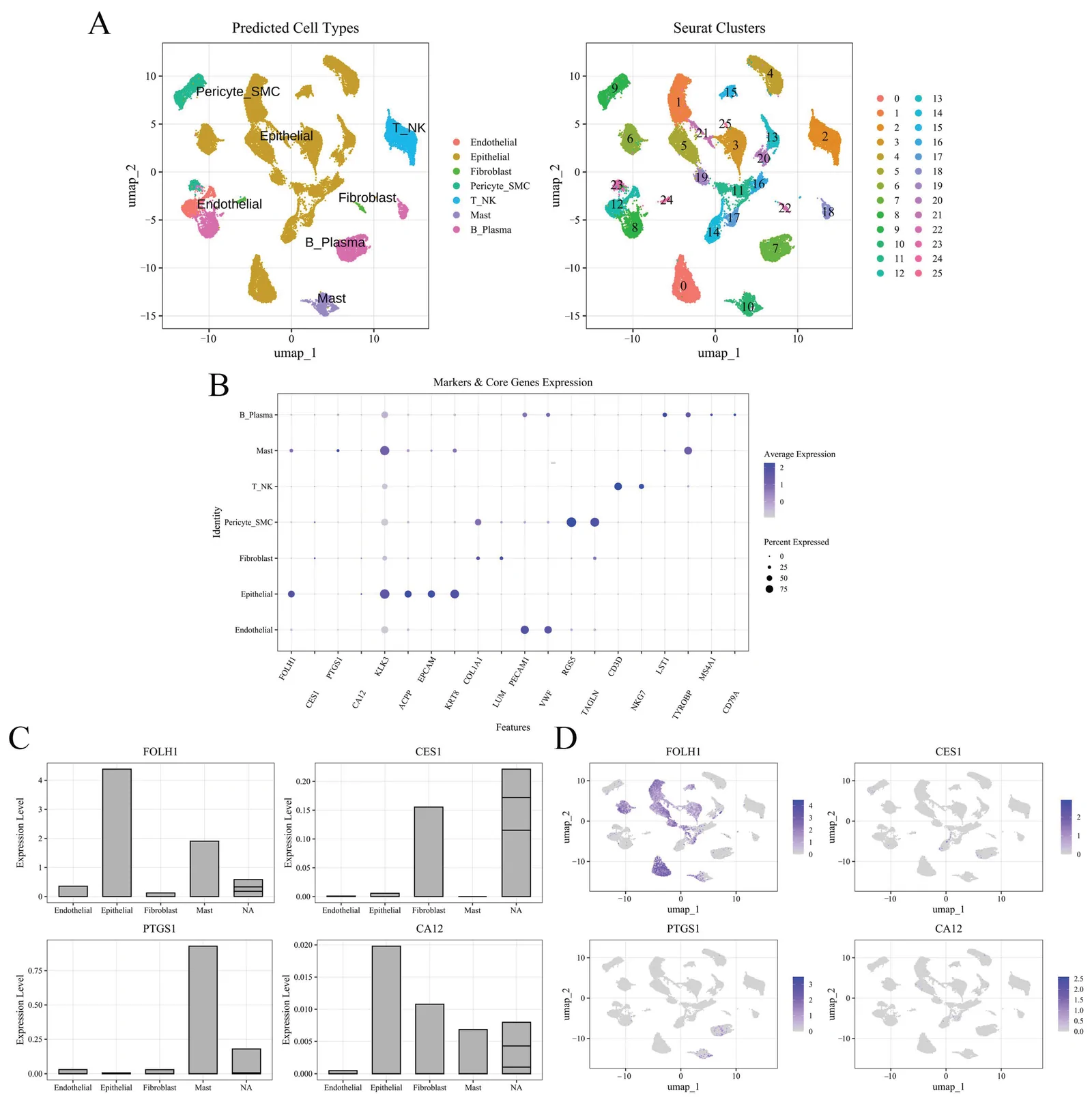
Single-cell RNA-seq characterization of core gene expression in the GSE141445 dataset: (**A**) UMAP visualization of predicted major cell types and Seurat-derived clusters. (**B**) Dot plot showing the expression patterns of canonical cell markers and core genes across annotated cell populations. (**C**) Box plots showing the cell type-associated expression levels of FOLH1, CES1, PTGS1, and CA12. (**D**) Feature plots displaying the spatial distribution of FOLH1, CES1, PTGS1, and CA12 expression on the UMAP embedding.

To further elucidate the expression patterns and cellular localization of core target genes within the prostate cancer tumor microenvironment (TME), single-cell RNA sequencing (scRNA-seq) analysis was performed using the GSE141445 dataset. After unsupervised clustering and UMAP dimensionality reduction, 26 transcriptionally distinct cell clusters (clusters 0–25) were identified (Figure 9A). These clusters were subsequently annotated and consolidated into seven major cell lineages, including epithelial cells, endothelial cells, fibroblasts, pericytes/smooth muscle cells (Pericyte_SMC), T/NK cells, mast cells, and B/plasma cells, based on canonical lineage-specific markers. The identities of the annotated cell populations were further validated using canonical marker genes (Figure 9B). Specifically, epithelial cells showed high expression of KLK3, ACPP, and EPCAM; fibroblasts were characterized by COL1A1 and LUM expression; endothelial cells prominently expressed PECAM1 and VWF; and immune and stromal populations were further supported by their corresponding marker profiles. Feature plot visualization and quantitative expression analysis further revealed distinct cell type-specific distribution patterns of the core target genes within the TME (Figure 9C,D). FOLH1, which encodes prostate-specific membrane antigen (PSMA), was predominantly enriched in epithelial cell populations, consistent with its close association with prostate tumor epithelial cells. CA12 also showed relatively higher expression in epithelial cells, suggesting a potential role in tumor parenchymal compartments. In contrast, CES1 expression was mainly detected in stromal cells, particularly fibroblasts, indicating its possible involvement in tumor-stroma interactions. Notably, PTGS1 showed preferential expression in mast cells, suggesting a potential role in immune cell-associated regulation within the prostate cancer microenvironment. Collectively, this single-cell transcriptomic analysis demonstrates the cellular heterogeneity of core target gene expression in the prostate cancer TME. These findings provide cellular-resolution evidence for understanding the potential biological functions of FOLH1, CA12, CES1, and PTGS1, thereby further highlighting their potential relevance as candidate targets for precision therapeutic strategies.

### 3.9. The Results of Spatial Transcriptome Analysis

**Figure 10:**
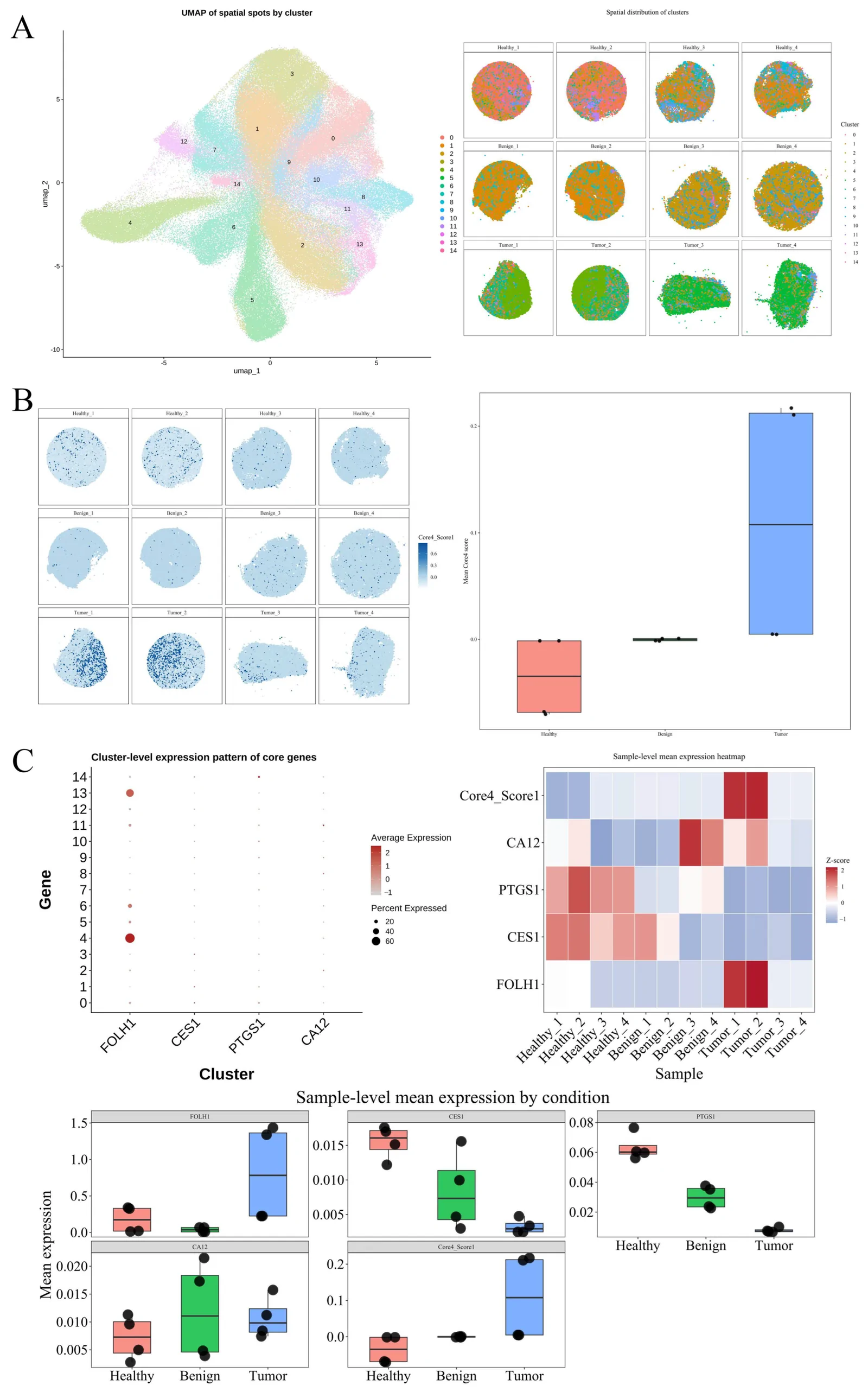
Spatial transcriptomic profiling of core gene signatures in the GSE181294 dataset: (**A**) UMAP visualization of spatial spots colored by cluster identity (**left**) and the spatial distribution of clusters across Healthy, Benign, and Tumor samples (**right**). (**B**) Spatial mapping of the Core4_Score1 signature across individual samples (**left**) and comparison of mean Core4_Score1 among different tissue conditions (**right**). (**C**) Cluster-level expression pattern of the core genes (FOLH1, CES1, PTGS1, and CA12) shown by dot plot (**left**), together with sample-level mean expression heatmap (**upper right**) and condition-wise comparison of mean expression levels for each core gene and Core4_Score1 (lower panel).

Spatial transcriptomic analysis was performed using the GSE181294 dataset to characterize the spatial distribution of DCPMU-related core genes in prostate cancer. As shown in Figure 10A, UMAP-based clustering identified 15 transcriptionally distinct spatial spot clusters that exhibited heterogeneous spatial organization across healthy, benign, and tumor prostate tissues. Compared with healthy and benign samples, tumor sections exhibited more pronounced regional enrichment of specific clusters, suggesting substantial spatial architectural remodeling during malignant progression. Spatial feature mapping further revealed distinct localization patterns of the core candidate gene signature. Core4 was defined as a four-gene DCPMU-associated signature comprising FOLH1, CES1, PTGS1, and CA12, whereas Core4_Score1 represented the relative coordinated expression of these genes within each spatial spot. As shown in Figure 10B, the Core4 module score exhibited stronger activation in tumor tissues than in healthy or benign tissues, with evident intratumoral heterogeneity. This observation suggests that the DCPMU-related transcriptional program is preferentially enriched within specific malignant spatial niches. To further quantify the spatial expression characteristics of individual core genes, cluster-level and sample-level analyses were performed. As shown in Figure 10C, FOLH1 displayed prominent enrichment in tumor-associated regions and exhibited focal spatial accumulation, whereas CES1 and PTGS1 were relatively enriched in non-tumor tissues and markedly reduced in tumor sections. CA12 displayed weaker and more sample-dependent spatial expression patterns. Consistently, sample-level heatmap and boxplot analyses further confirmed increased FOLH1 expression and elevated Core4 scores in tumor samples, together with decreased CES1 and PTGS1 expression and variable CA12 expression across samples. Collectively, these spatial transcriptomic findings indicate that DCPMU-related core genes undergo marked spatial reprogramming in prostate cancer. Tumor-enriched activation of the Core4 signature, together with FOLH1-dominant spatial localization, highlights a potential DCPMU-associated malignant spatial ecosystem and provides spatial-level evidence supporting the biological relevance of these core genes in prostate cancer progression.

### 3.10. The Results of Virtual Disturbance Analysis

**Figure 11:**
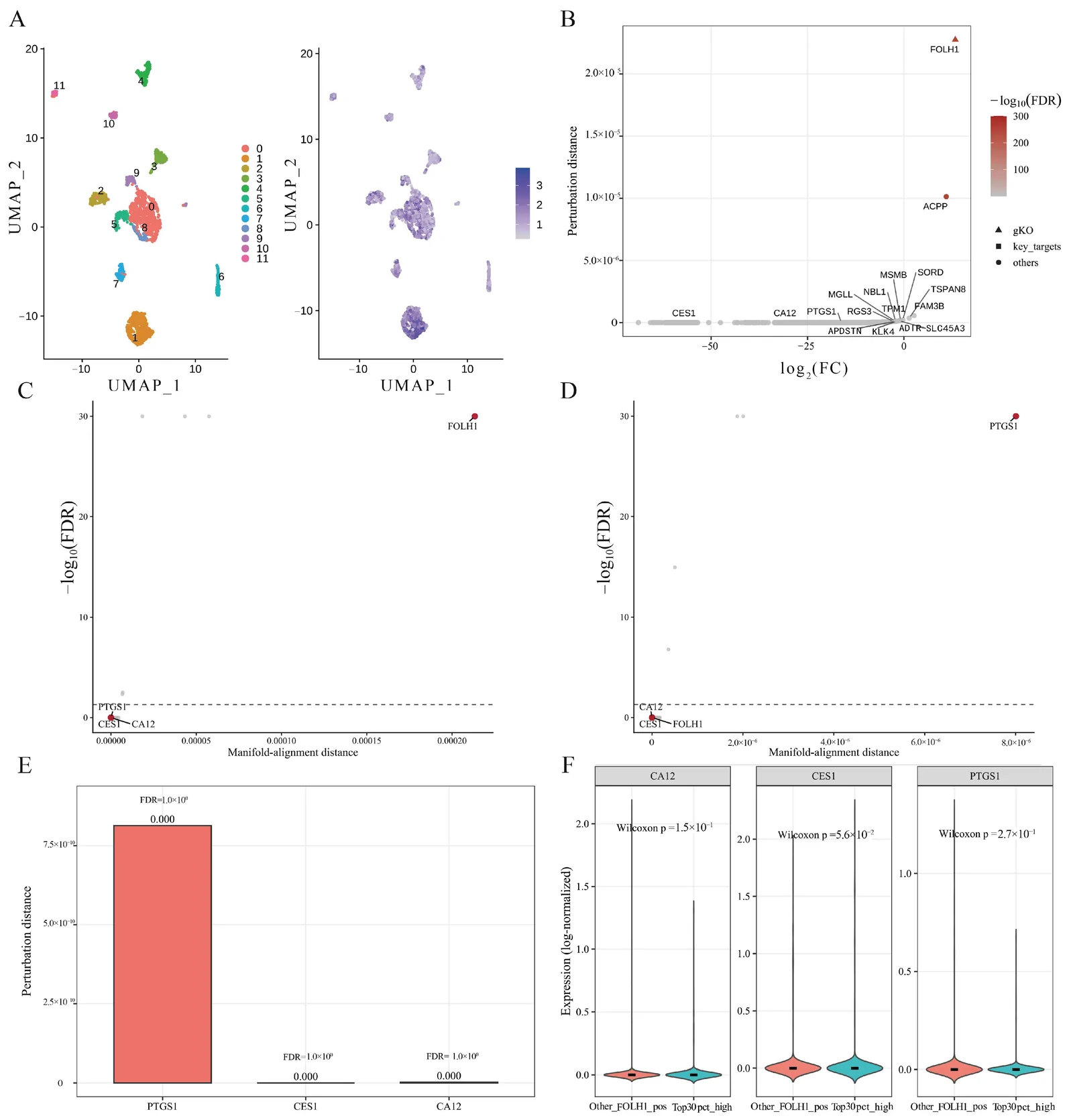
Virtual perturbation analysis of FOLH1 in the GSE181294 single-cell dataset: (**A**) UMAP visualization of target cell clusters selected for virtual perturbation analysis and UMAP feature plot showing heterogeneous FOLH1 expression across target cells. (**B**) Global perturbation landscape of differentially regulated genes after virtual FOLH1 knockout inferred by scTenifoldKnk. (**C**) Differential-regulation landscape following virtual FOLH1 knockout inferred by scTenifoldKnk, with FOLH1, PTGS1, CES1, and CA12 highlighted for comparison. (**D**) Differential-regulation landscape following parallel virtual PTGS1 knockout from the same tensor-denoised WT regulatory network, highlighting reciprocal and cross-KO responses of FOLH1, PTGS1, CES1, and CA12. (**E**) Predicted perturbation effects of FOLH1 knockout on DCPMU-related downstream candidates, including PTGS1, CES1, and CA12. (**F**) Observed expression patterns of CA12, CES1, and PTGS1 in FOLH1-defined tumor cell populations.

To further infer the potential regulatory roles of DCPMU-related core genes in prostate cancer, virtual perturbation analysis was performed on the GSE181294 spatial transcriptomic dataset using scTenifoldKnk. As shown in Figure 11A, UMAP projection of target cells revealed heterogeneous FOLH1 expression across tumor-associated cell clusters, suggesting spatially restricted activity within malignant regions. After in silico knockout of FOLH1, global perturbation profiling was performed to evaluate downstream transcriptional responses. As shown in Figure 11B, FOLH1 was identified as the most strongly perturbed node, accompanied by marked perturbation of prostate cancer-associated genes, including ACPP and MSMB. These results suggest that FOLH1 perturbation may exert broad regulatory effects on the prostate cancer transcriptional network. Virtual knockout of FOLH1 induced a distinct network perturbation pattern, with FOLH1 representing the most strongly affected node in terms of manifold-alignment distance and statistical significance (Figure 11C). Several additional genes also exhibited significant differential regulation, whereas the predefined candidates PTGS1, CES1, and CA12 showed minimal perturbation and remained below the FDR < 0.05 threshold. Parallel virtual knockout of PTGS1 yielded a comparable pattern, with PTGS1 emerging as the dominant perturbed node and a subset of additional genes displaying significant network-level responses (Figure 11D). In contrast, FOLH1, CES1, and CA12 showed limited perturbation following PTGS1 knockout. Together, these results indicate that FOLH1 and PTGS1 each influence the inferred tumor epithelial regulatory network, while their reciprocal effects and their effects on CES1 and CA12 appear limited under the present virtual perturbation framework. Among the DCPMU-related candidate genes, PTGS1 showed the strongest perturbation response after FOLH1 knockout, whereas CES1 and CA12 exhibited comparatively weaker responses (Figure 11E). This finding suggests that PTGS1 may represent a major downstream node showing a strong predicted response to FOLH1 perturbation. Furthermore, as shown in Figure 11F, the expression differences in CA12, CES1, and PTGS1 between FOLH1-positive tumor cells and the top 30% FOLH1-high tumor cells were relatively modest. These findings indicate that the predicted regulatory influence of FOLH1 may reflect network-level dependency rather than simple co-expression. Collectively, these results suggest that FOLH1 may represent a candidate network-associated node within the prostate cancer transcriptional program. PTGS1 showed the most prominent predicted response among the examined candidates, suggesting a potential FOLH1-PTGS1 network relationship that requires further experimental validation.

## 4. Discussion

DCPMU, also known as desmethyl-diuron or 1-(3,4-dichlorophenyl)-3-methylurea, is a major transformation product of diuron generated through environmental and biological N-demethylation. Owing to the extensive agricultural and antifouling applications of diuron, DCPMU has been recognized as an environmentally relevant contaminant rather than a negligible degradation by-product [26]. Monitoring studies and regulatory documents have reported the occurrence of diuron and its degradates, including DCPMU, in groundwater, surface water, and coastal aquatic systems [27]. For example, the California Department of Pesticide Regulation has documented detections of diuron, 3,4-dichloroaniline, and DCPMU in California groundwater, including domestic wells, and established a human health reference level of 100 ppb for diuron residues and related degradates in groundwater screening. Similarly, the U.S. Environmental Protection Agency has validated analytical methods for quantifying diuron, DCPMU, and related degradates in drinking water and surface water at low microgram-per-liter levels, supporting their relevance for environmental surveillance. Evidence from agricultural and coastal regions further indicates that diuron-derived contamination is not geographically restricted. Diuron and its metabolites, including DCPMU and DCPU, have been detected in coastal seawater in China, while studies in Brazilian agricultural settings have highlighted the environmental persistence of diuron and its conversion into toxic metabolites [28]. Importantly, regulatory frameworks increasingly incorporate diuron metabolites into risk assessment. The European Food Safety Authority defines relevant residues as the sum of diuron and metabolites containing the 3,4-dichloroaniline moiety, expressed as diuron, and California regulations list diuron-containing products as chemicals with groundwater-pollution potential. Although direct epidemiological evidence linking DCPMU to prostate cancer remains limited, its environmental persistence, detectable occurrence, and regulatory recognition support the toxicological importance of investigating DCPMU-associated molecular perturbations in prostate carcinogenesis.

The seven DCPMU-related core genes identified through differential expression analysis and external validation in this study, including APOBEC3G, SCGB1A1, PTGS1, CA12, CES1, FOLH1, and NOS1, suggest that DCPMU may influence prostate cancer through coordinated alterations in genomic stability, inflammatory metabolism, hypoxic adaptation, xenobiotic handling, and prostate lineage-associated signaling. APOBEC3G, a member of the APOBEC3 cytidine deaminase family, may represent a DNA/RNA-editing and innate immune-related axis; APOBEC3-mediated mutagenesis has been linked to tumor heterogeneity, immune modulation, and therapeutic resistance, with emerging evidence in prostate cancer models [29]. In contrast, SCGB1A1 has recently been reported to exert tumor-suppressive effects in prostate cancer by inhibiting proliferation, migration, invasion, and epithelial-mesenchymal transition, partly through modulation of MAPK signaling, suggesting that SCGB1A1 dysregulation may compromise epithelial homeostasis [30]. PTGS1, which encodes cyclooxygenase-1 (COX-1), catalyzes prostaglandin synthesis and participates in angiogenesis, inflammation, and tumor-associated proliferative processes, indicating a potential mechanism by which DCPMU may reshape inflammatory lipid signaling [31]. CA12, a hypoxia-inducible carbonic anhydrase, regulates intracellular pH and extracellular acidification under hypoxic stress, thereby supporting tumor cell survival and invasion within acidic microenvironments [32]. CES1 is involved in xenobiotic metabolism and lipid/cholesterol homeostasis, and genetic evidence has linked CES1 variants to biochemical recurrence-free survival in prostate cancer, highlighting its relevance to metabolic detoxification and disease progression [33]. Notably, FOLH1, which encodes prostate-specific membrane antigen (PSMA), is not only an established clinical imaging and therapeutic target but is also increasingly recognized as a functional regulator of prostate cancer biology and intratumoral heterogeneity [34]. Finally, NOS1 implicates nitric oxide signaling, which has been associated with DNA damage, prostate cancer stem-like phenotypes, therapeutic resistance, and metastatic potential [35]. Collectively, these findings support a model in which DCPMU-associated molecular perturbations may contribute to prostate carcinogenesis through interconnected effects on mutational stress, epithelial differentiation, inflammatory prostaglandin signaling, hypoxia-pH adaptation, xenobiotic metabolism, PSMA-related prostate lineage programs, and nitric oxide-mediated tumor progression.

Molecular docking analysis indicated that DCPMU exhibited favorable binding to the seven core targets, with binding energies ranging from −5.536 to −7.407 kcal/mol. Based on docking affinity, biological interpretability, prostate cancer relevance, and consistency with transcriptomic evidence, PTGS1, CA12, CES1, and FOLH1 were prioritized for molecular dynamics (MD) simulations. PTGS1, which encodes cyclooxygenase-1 (COX-1), is a key mediator of prostaglandin biosynthesis and may link DCPMU exposure to inflammatory lipid signaling, angiogenesis, and tumor progression [36]. CA12, a hypoxia-responsive carbonic anhydrase, contributes to pH homeostasis and extracellular acidification, thereby supporting tumor adaptation and invasion under hypoxic stress [37]. CES1 is involved in xenobiotic metabolism, lipid turnover, and cholesterol homeostasis, highlighting its potential relevance to toxicant handling and prostate cancer progression [38]. FOLH1, which encodes prostate-specific membrane antigen (PSMA), is a well-established biomarker and therapeutic target in prostate cancer and is closely associated with malignant epithelial identity and intratumoral heterogeneity. Although NOS1 may be involved in nitric oxide signaling, current evidence more strongly supports a broader NOS/NO axis than a NOS1-specific mechanism; therefore, NOS1 was considered a secondary candidate [39]. MD simulations further revealed distinct dynamic behaviors among the four selected DCPMU-protein complexes. One complex remained comparatively stable throughout the simulation, as indicated by low and convergent RMSD values and a concentrated low-energy basin in the free energy landscape, consistent with a relatively favorable binding mode. By contrast, the other complexes displayed marked ligand-complex RMSD fluctuations, including intermittent sharp increases, whereas protein backbone deviations remained generally low. This pattern suggests that the proteins retained overall structural integrity, while the observed instability primarily arose from ligand reorientation, local migration within the binding pocket, or partial dissociation from the initial docking pose. RMSF profiles further indicated that the major fluctuations were concentrated in flexible loop and terminal regions rather than in the structured protein core. Such behavior may be attributable to local pocket flexibility, solvent exposure, and dynamic disruption and reformation of hydrogen-bonding and hydrophobic interactions. Overall, these findings suggest that DCPMU-target interactions are dynamically plausible; however, complexes exhibiting large RMSD oscillations should be interpreted with caution.

By integrating single-cell transcriptomic, spatial transcriptomic, and virtual perturbation analyses, this study provides a multiscale view of DCPMU-associated molecular alterations in prostate cancer. Single-cell analysis revealed marked cellular heterogeneity, identifying epithelial, endothelial, fibroblast, pericyte/smooth muscle, T/NK, mast, and B/plasma cell populations. Among the DCPMU-related core genes, FOLH1 was predominantly enriched in epithelial cells, consistent with its established prostate epithelial lineage specificity [40], whereas CES1, PTGS1, and CA12 showed more restricted or non-uniform expression across immune, stromal, and epithelial compartments. These findings suggest that DCPMU-associated transcriptional effects may involve both tumor-intrinsic and microenvironmental components. Spatial transcriptomic analysis further supported this compartmentalized pattern, showing tumor-enriched FOLH1 expression and elevated Core4 module scores in malignant regions, whereas CES1 and PTGS1 were relatively reduced in tumor sections, and CA12 exhibited sample-dependent spatial variation. These results indicate that the DCPMU-related signature is not uniformly distributed but is spatially organized within prostate cancer tissues, with FOLH1-dominant activation marking malignant epithelial niches. Importantly, parallel virtual knockout analysis using scTenifoldKnk, performed from the same tensor-denoised WT regulatory network, revealed distinct network-level differential-regulation patterns following perturbation of either FOLH1 or PTGS1. FOLH1 exhibited high WT-network centrality, supporting its potential role as a regulatory hub; however, FOLH1 knockout produced only limited perturbation of PTGS1, CES1, and CA12, while PTGS1 knockout similarly showed little reciprocal effect on FOLH1 or on CES1 and CA12. These findings argue against a simple linear FOLH1-PTGS1/CES1/CA12 regulatory axis and instead suggest that DCPMU-associated epithelial reprogramming may involve broader and distributed regulatory-network remodeling [41]. Thus, FOLH1 may represent a tumor-enriched network node whose functional effects are mediated through wider regulatory programs rather than direct regulation of the selected DCPMU-related candidates.

However, several limitations should be acknowledged. This study was mainly based on computational inference and public transcriptomic datasets lacking direct DCPMU exposure information. Therefore, causal relationships between DCPMU exposure and prostate cancer progression cannot be established. In addition, sample size and dataset heterogeneity may affect generalizability. Further experimental validation and exposure-based epidemiological studies are required.

## 5. Conclusions

This study integrated network toxicology, molecular modeling, single-cell transcriptomics, spatial transcriptomics, and virtual perturbation analysis to investigate the potential effects of DCPMU on prostate cancer. The results identified four validated core genes and suggested that DCPMU-associated molecular perturbations may involve FOLH1-dominant epithelial remodeling, PTGS1-related inflammatory regulation, CA12-mediated hypoxia-pH adaptation, and CES1-linked xenobiotic metabolism. Spatial transcriptomic and virtual perturbation analyses further indicated that FOLH1 may represent a key regulatory node within malignant epithelial niches. Collectively, these findings suggest that DCPMU may influence prostate cancer progression through coordinated modulation of tumor cell states and microenvironmental interactions, highlighting the need for further experimental validation.

## Figures and Tables

**Table 1:** Results of the free energy calculations (kcal/mol).

Energy Components	CA12	CES1	FOLH1	PTGS1
VDWAALS	−103.855	4.460	−156.628	−92.713
EEL	−50.606	10.728	−28.577	−27.536
EPB	101.097	−0.044	95.845	71.664
ENPOLAR	−13.183	10.728	−14.185	−11.874
GGAS	−154.461	15.188	−185.205	−120.249
GSOLV	87.915	−0.061	81.660	59.790
Binding	−66.546	4.388	−103.545	−60.459

## Data Availability

The datasets analyzed in this study are publicly available from The Cancer Genome Atlas (TCGA) and the Gene Expression Omnibus (GEO). The corresponding dataset accession numbers and data sources are provided in the Section 2 of the manuscript.

## Abbreviations

The following abbreviations are used in this manuscript:

DCPMU	3-(3,4-Dichlorophenyl)-1-methylurea
DEGs	Differentially expressed genes
PPI	Protein–protein interaction
KEGG	Kyoto Encyclopedia of Genes and Genomes
GO	Gene Ontology
RMSD	Root mean square deviation
RMSF	Root mean square fluctuation
VDWAALS	Van der Waals energy
EEL	Electrostatic energy
EPB	Poisson–Boltzmann polar solvation energy
ENPOLAR	Non-polar solvation energy
GGAS	Gas-phase/solvation-related energy combination
GSOLV	Solvation free energy
